# Towards stunting eradication in Indonesia: Time to invest in community health workers

**DOI:** 10.1002/puh2.108

**Published:** 2023-07-21

**Authors:** Adriana Viola Miranda, Trio Sirmareza, Ryan Rachmad Nugraha, Maritta Rastuti, Habibi Syahidi, Rindang Asmara, Zack Petersen

**Affiliations:** ^1^ Global Health Focus Asia Bandung Indonesia; ^2^ 1000 Days Fund Jakarta Indonesia; ^3^ Department of Family Medicine and Population Health University of Antwerp Antwerp Belgium

**Keywords:** access, child health, community health workers, home‐based counseling, Indonesia, nutrition, public health intervention, stunting

## Abstract

In Indonesia, home to the fourth‐largest children‐under‐five population globally, the stunting prevalence is among the highest in Asia. The detrimental and irreversible impacts of stunting warranted the government to aim for stunting eradication by 2030, with community health workers (CHWs) at the forefront of the program. With over 1.5 million Indonesian CHWs conducting monthly growth monitoring and counseling, the critical importance and cost‐effectiveness of CHWs in stunting management are clear. However, several implementation challenges continue to hinder their maximum potential. This includes unclear recruitment processes, the nonprofessional status of CHWs, unclear incentive schemes, research and funding constraints, and unclear role of stakeholders. This commentary examines the current involvement of CHWs in the Indonesian stunting program, the remaining issues, and recommendations to mitigate the identified challenges. Improvements in the recruitment process, recognition, and incentivization system, as well as cross‐sectoral collaboration, particularly in evidence creation and policy‐research continuum, are crucial in improving the current program and achieve the goal of stunting eradication.

## Graphical abstract

Graphical Abstract: This commentary examined the current involvement of community healthcare workers (CHWs) in the Indonesian stunting program, the remaining challenges, and provided recommendations to improve the current program and help achieve the goal of stunting eradication.

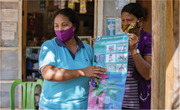

## INTRODUCTION

Stunting remains a major issue globally despite decreasing prevalence each year. In 2020, 22% of children globally are estimated to be stunted [1]. In Indonesia, home to the fourth‐largest children‐under‐five population globally [2], the latest reported stunting prevalence (2022) is at 21.6%, affecting more than four million children nationally [3]. It is among the highest prevalence in Asia. This is alarming as stunting has detrimental long‐term effects for both the affected individuals and the socioeconomic development of their society. Stunted growth in children, with its irreversible nature, causes impaired cognitive and physical development, as well as increased risk of poor health status and degenerative diseases, reducing their productive capacity [4].

Recognizing the importance of stunting, Indonesia has a goal to eradicate stunting by 2030, with a mid‐term goal of reducing the prevalence to 14% by 2024. The Indonesian government outlined several strategies to achieve this goal, mainly through conducting awareness campaigns and accessible healthcare services. This is in response to the nature of factors associated with stunting in Indonesia that are largely related to socioeconomic status, such as poor access to healthcare, poor sanitation status, and low maternal education. The government puts community health workers (CHWs) at the forefront of its strategies: working in a program called *Posyandu* (*pos pelayanan terpadu* or integrated healthcare center), CHWs are mandated to conduct information dissemination, periodic nutrition assessments, and reporting to the authorities [5]. However, with only 1 year before 2024, more efforts are needed: A projection showed that the current reduction rate needs to be doubled to achieve the goal on time [6]. This commentary aims to examine the current stunting eradication efforts in Indonesia and the role of CHWs, as well as provide evidence‐based recommendations.

## CHWs: THE BACKBONE OF STUNTING MANAGEMENT

Within the Indonesian health system, *Posyandu* are low‐level health services below the government‐owned primary care facilities called the community health centers (CHCs). A *Posyandu* is a program with at least five tables setup: Each table serves as a place to provide a specific maternal and child health service, from registration, growth measurement, to counseling. Every *Posyandu* serves approximately 100 children‐under‐five and 700 people in its local community. It is generally managed by three to five CHWs, each attending one *Posyandu* table [7].

As of 2019, there are 302,150 *Posyandu* and over 1.5 million CHWs working on maternal and child health programs in Indonesia [8]. These CHWs are appointed by the village office from their communities to help government programs in a culturally sensitive manner. Although CHWs are not only tasked with managing the *Posyandu*, but also other primary healthcare services, such as noncommunicable diseases and first aid services [7], CHWs have their largest network deployed in *Posyandu* [8]. To conduct their tasks, CHWs are regulated and financed by village offices. CHWs also collaborate with the CHCs in creating awareness and assessment programs, assessing nutritional status and providing nutritional counseling for mothers and their children. Aside from delivering nutritional intervention, CHWs also serve as the bridge between their communities and healthcare professionals. They often conduct home visits and provide simple health advice for their communities [5]. Despite the abundant task, the incentive schemes for CHWs are unclear. Many CHWs work on a voluntary basis, receiving only reimbursement for transportation fees, and receive minimal training [7].

The known impact of CHWs on stunting management cannot be overstated. A study showed that at 90% coverage, ten nutrition‐specific interventions could reduce stunting prevalence by 20% [9]. In Indonesia, seven of these interventions are the main tasks of CHWs in *Posyandu* [5], including counseling, health promotion, and various nutrition supplementation for pregnant women and children‐under‐five [9]. Furthermore, recent studies in Bangladesh and Kenya show that home‐based counseling conducted by CHWs significantly reduced stunting prevalence [10, 11]. The role of CHWs is even more important in remote settings where resources are scarce and access is limited. In Guatemala, CHWs can be accounted for delivering nutritional education and services for indigenous communities [12]. As CHWs are members of their communities, they know the local context well enough to effectively do outreach, allowing lasting results and impacts. Furthermore, various studies have shown that CHWs intervention is more cost‐effective compared to facility‐based programs [13].

**FIGURE 1:**
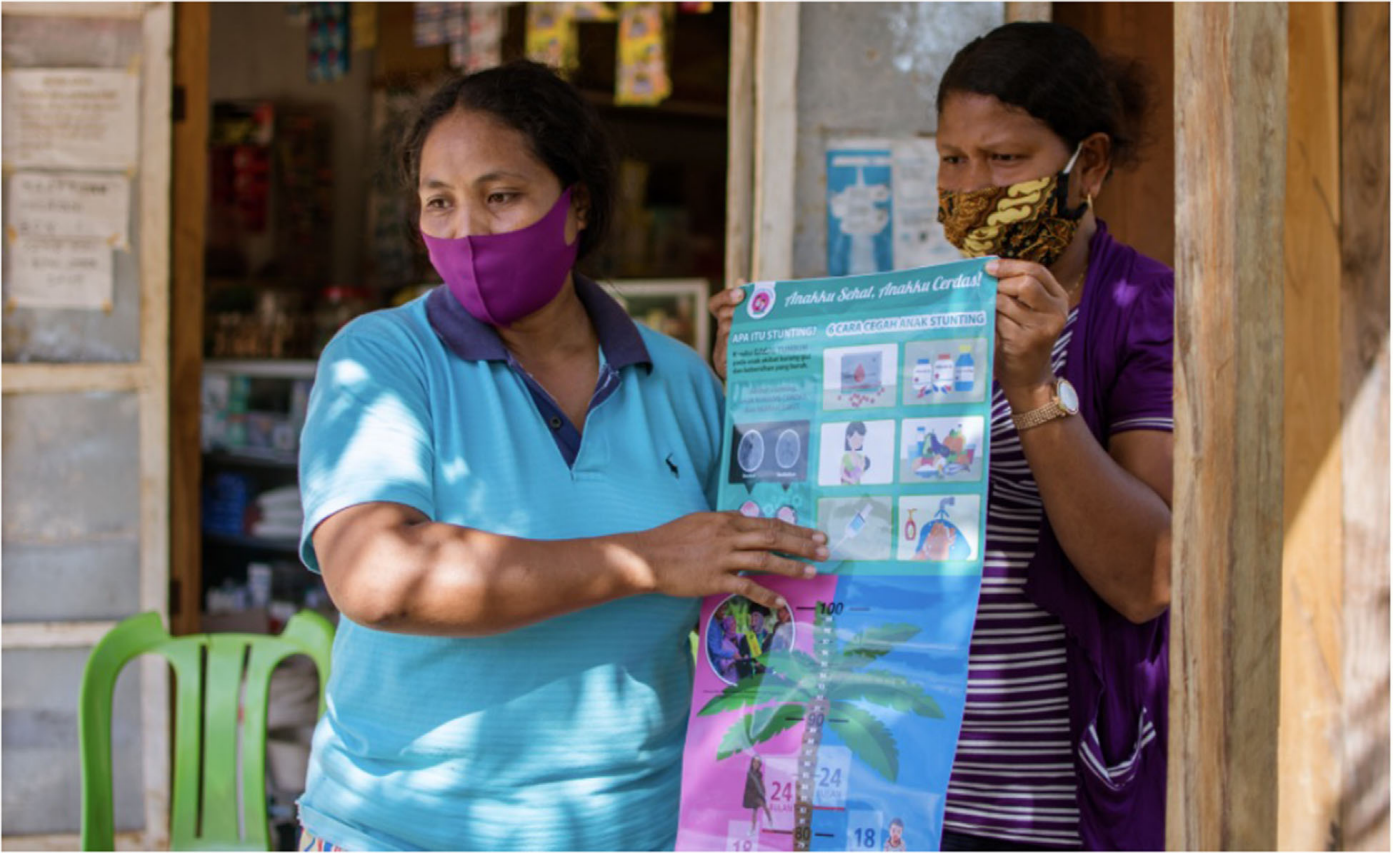
A group of community health workers (CHWs) in Eahun, Rote Ndao, East Nusa Tenggara, conducting stunting prevention education and counseling using the 1000 Days Fund smart chart.

In Indonesia, the sheer number of CHWs creates a massive opportunity to utilize these strengths, particularly for stunting prevention and management. The crucial role of CHWs is supported by our own observation in the Indonesian province of East Nusa Tenggara, where the 1000 Days Fund has been delivering a package of stunting interventions since 2019. The interventions entail delivering home‐based height monitoring and educational smart charts to families, as well as training programs for CHWs (Figure 1). The intervention is actively given to over 16,500 families with children‐under‐five across four regencies (the term for “cities” in Indonesian rural settings) with the highest stunting prevalence in the province. This covers 25.65% of all families in the regencies. In Rote, an island in one of the regencies, we have trained 97% (1,697) of its CHWs. Our interventions have increased CHWs activities over the years: A study we conducted found that certified CHWs are 23 times more likely to conduct home visits compared to noncertified CHWs [14]. The 2022 stunting prevalence in these regencies was reduced by 11–19 percentage points compared to 2019, a higher reduction compared to other regencies in the province [3, 15].

## REMAINING CHALLENGES

Despite the importance of CHWs in stunting management, several issues continue to hinder their maximum potential. In general, we observed that the recruitment criteria for CHWs are unclear, leading to unstandardized capacity among CHWs. In some regions, CHWs recruitment remains lacking in continuity and sustainability principles: CHWs are often replaced as new village heads are appointed due to reasons unrelated to their work [16]. This leads to the need to retrain CHWs, thus requiring more funds and resources.

Furthermore, the status of CHWs as nonprofessional workers leads to quality and recruitment issues. Although the Ministry of Health mandates local governments to train CHWs, in practice, they receive minimal training and supervision. Most of the training programs were given by nongovernmental organizations (NGOs) [17]. This resulted in varying knowledge and practices among the CHWs [17, 18]. Some CHWs have expressed their disappointment toward the lack of engagement from the government [17]. It is concerning considering the Indonesian stunting program relies heavily on CHWs at its forefront, both in terms of stunting prevention and reporting. CHWs also do not have a clear incentive scheme: CHWs’ highly preferred incentives, training and recognition, are limited at best, and the income of CHWs relies on “gifts” from stakeholders, with various disbursement mechanisms according to local policies [17, 19]. Consequently, the recruitment of new CHWs has stagnated, with many younger people unwilling to work as CHWs [18]. Shortage of CHWs is increasingly reported in Indonesia [20], particularly as the annual dropout rates for CHWs were reported to be between 20% and 30% [7].

The issues above are inextricably linked to the financial sustainability of CHWs. Although it is clear that investing in CHWs is cost‐effective [13], the Indonesian CHWs programs continue to be hindered by funding constraints and the lack of political support [20]. Without sufficient funds, it is challenging to create effective training and incentivization programs. This may be related to the fact that evidence on CHWs in the Indonesian context is still limited [19]: How can local stakeholders ascertain that investing in CHWs is promising if the number of Indonesian‐based studies remains minimal? Research projects, such as cost‐effectiveness studies, are needed to enable long‐term investment in CHWs, particularly to support the sustainability of CHWs.

The unclear role of stakeholders further exacerbates the dire situation of CHWs. In Indonesia, stunting is a strategic issue that involves a lot of ministries and stakeholders. Although the roles of these stakeholders were outlined in government documents, from our observations, confusion regarding the implementation of these roles remains a major issue. Although per the documents, the regulation of CHWs falls under the jurisdiction of the village office [5], CHCs are still widely believed to be in the charge of supervising the CHWs. These two stakeholders are regulated by different ministries (Ministry of Village and Ministry of Health), further complicating the awareness and collaborative efforts on this issue.

## RECOMMENDATIONS

Governments of Indonesia and globally should invest in CHWs so that their potential can be maximized, given their important roles in the health systems. Transparent recruitment and recognition are the crucial starting points: The recognition of CHWs should start by creating a clear certification system, which has proven effective in the Indonesian province of West Nusa Tenggara [16].

It is also crucial to create an incentive system that motivates CHWs to perform efficiently. The government should follow evidence‐based interventions in incentivizing CHWs through both monetary and nonmonetary measures. Training programs should be prioritized as nonmonetary incentives, as aside from being the CHWs‐preferred incentives, adequate training will also help CHWs deliver service more efficiently [17, 18]. Monetary incentives may also encourage CHWs to be more active in their communities; our local experience in two East Nusa Tenggara districts shows that CHWs receiving monetary incentives are twice as likely to initiate home‐based stunting education within their communities compared to non‐incentivized CHWs.

Furthermore, to continuously improve CHWs interventions and allow for more financial commitments from stakeholders, evidence creation should be prioritized. The government should collaborate with researchers and universities and create engagement opportunities for field workers, such as NGOs, to conduct research projects with these researchers. The knowledge‐to‐policy (K2P) approach should be used to ensure that future policies and interventions are informed by the real‐world evidence obtained from these research programs. The implementation of a policy‐research continuum should be considered. The continuum includes five phases: early assessments, policy surveillance, implementation research, policy rating, and impact research [21].

**FIGURE 2:**
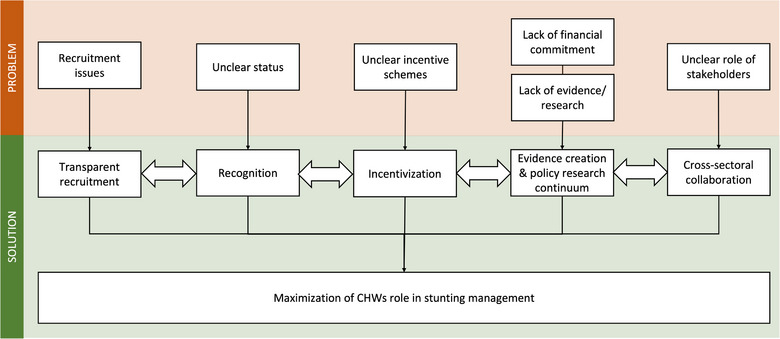
Pathway for evidence‐based stunting management.

In working on these recommendations, close collaboration with locally led NGOs and implementers is highly recommended to enable knowledge transfer and sustainability. Therefore, each stakeholder should be clearly informed regarding their roles within the CHWs program, such as by conducting interministerial awareness programs. Figure 2 summarizes these recommendations required to maximize CHWs’ potential in stunting management. Note that each recommendation is interconnected with the other. An effective policy‐research continuum informed by real‐world evidence will pave the way for better recognition and incentivization of CHWs, thus improving the stunting management program. This improvement will then produce more evidence on CHWs that should be addressed to maximize the program's impact.

## CONCLUSION

The important role of CHWs at the forefront of stunting management in Indonesia should be recognized by all related stakeholders. Adequate support should be given by developing frequent training programs, clear incentive systems, and quality assurance. These efforts, which should be achieved through multisectoral collaboration, are the keys to maximizing CHWs’ potential and stunting eradication in Indonesia.

## AUTHOR CONTRIBUTIONS

*Conceptualization; data curation; writing—original draft; writing—review and editing*: Adriana Viola Miranda. *Conceptualization; data curation; supervision; writing—review and editing*: Trio Sirmareza. *Conceptualization; data curation; writing—review and editing*: Ryan Rachmad Nugraha. *Data curation; writing—review and editing*: Maritta Rastuti, Habibi Syahidi. *Writing—review and editing*: Rindang Asmara. *Supervision; writing—review and editing*: Zack Petersen.

## CONFLICT OF INTEREST STATEMENT

AVM and RRN are editorial board members of the journal. They were excluded and blinded from all stages of the peer review of this manuscript.

## FUNDING INFORMATION

MAC3 Impact Philanthropies

## Data Availability

No database or primary data was used in writing the paper.

## References

[1] World Health Organization . Stunting Prevalence among Children under 5 Years of Age (%) (Model‐based Estimates). World Health Organization; 2020. Accessed February 10, 2023. https://www.who.int/data/gho/data/indicators/indicator‐details/GHO/gho‐jme‐stunting‐prevalence

[2] UNICEF . How Many? – UNICEF Data. UNICEF. Accessed February 10, 2023. https://data.unicef.org/how‐many/

[3] Indonesian Ministry of Health . Buku saku hasil studi status gizi Indonesia (SSGI) Tahun 2022 [Guidebook of the Results of Nutritional Status in Indonesia in 2022]. Indonesian Ministry of Health; 2022. Published online December 2022. Accessed February 14, 2023. https://promkes.kemkes.go.id/download/grep/files52434Buku%2520Saku%2520SSGI%25202022%2520rev%2520210123.pdf

[4] World Health Organization . Global Nutrition Targets 2025: Stunting Policy Brief. World Health Organization; 2014. Published online December 30, 2014. Accessed January 10, 2023. https://www.who.int/publications/i/item/WHO‐NMH‐NHD‐14.3

[5] The National Team for the Acceleration of Poverty Reduction . Strategi nasional percepatan pencegahan anak kerdil (stunting) periode 2018–2024 [National Strategy for Stunting Prevention Acceleration 2018‐2024]. The National Team for the Acceleration of Poverty Reduction; 2018. Published online 2018. Accessed January 9, 2023. https://tnp2k.go.id/filemanager/files/Rakornis%202018/Stranas%20Percepatan%20Pencegahan%20Anak%20Kerdil.pdf

[6] Lembaga Demografi FEB UI . Angka stunting Indonesia masih tertinggi kedua setelah Papua Nugini di Asia‐Pasifik [The Stunting Rate in Indonesia is the Second Highest after Papua Nugini in Asia‐Pacific region]. Lembaga Demografi FEB UI; 2022. Published August 4, 2022. Accessed February 15, 2023. https://ldfebui.org/angka‐stunting‐indonesia‐masih‐tertinggi‐kedua‐setelah‐papua‐nugini‐di‐asia‐pasifik/

[7] Oendari A , Rohde J . Indonesia's community health workers (kaders). In: Perry HB , ed. Health for the People: National Community Health Programs from Afghanistan to Zimbabwe. USAID/Jhpiego; 2020:149‐164. https://chwcentral.org/wp‐content/uploads/2021/11/Health_for_the_People_Natl_Case%20Studies_Oct2021.pdf. accessed May 3, 2023. Available from.

[8] Health Research and Development Agency, Indonesian Ministry of Health . Laporan riset fasilitas kesehatan (Rifaskes) 2019: Puskesmas [Health Facilities Research Report 2019: Community Health Centers]. Health Research and Development Agency, Indonesian Ministry of Health; 2019. Published online 2019. Accessed February 15, 2023. https://www.litbang.kemkes.go.id/laporan‐hasil‐riset‐fasilitas‐kesehatan/

[9] Bhutta ZA , Das JK , Rizvi A , et al. Evidence‐based interventions for improvement of maternal and child nutrition: what can be done and at what cost? Lancet North Am Ed. 2013;382(9890):452‐477. doi:10.1016/S0140-6736(13)60996-4 23746776

[10] Mistry SK , Hossain MdB , Arora A . Maternal nutrition counselling is associated with reduced stunting prevalence and improved feeding practices in early childhood: a post‐program comparison study. Nutr J. 2019;18:47. doi:10.1186/s12937-019-0473-z 31455363 PMC6712751

[11] Nyamasege CK , Kimani‐Murage EW , Wanjohi M , Kaindi DWM , Wagatsuma Y . Effect of maternal nutritional education and counselling on children's stunting prevalence in urban informal settlements in Nairobi, Kenya. Public Health Nutr. 2021;24(12):3740‐3752. doi:10.1017/S1368980020001962 32693855 PMC7611536

[12] Perry HB , Stollak I , Llanque R , et al. Reducing inequities in maternal and child health in rural Guatemala through the CBIO+ Approach of Curamericas: 4. Nutrition‐related activities and changes in childhood stunting, wasting, and underweight. Int J Equity Health. 2023;21(S2):197. doi:10.1186/s12939-022-01756-8 36855101 PMC9973244

[13] Vaughan K , Kok MC , Witter S , Dieleman M . Costs and cost‐effectiveness of community health workers: evidence from a literature review. Hum Resour Health. 2015;13(1):71. doi:10.1186/s12960-015-0070-y 26329455 PMC4557864

[14] 1000 Days Fund . 1000 Days Fund Annual Report 2021. 1000 Days Fund; 2022. [Accessed May 3, 2023]. Available from: https://1000daysfund.org/our‐approach/insights‐impact/

[15] Izwardy D . Studi status gizi balita terintegrasi Susenas 2019 [Susenas‐integrated nutritional status study of children‐under‐five 2019] . Published online February 20, 2020. https://persi.or.id/wp‐content/uploads/2020/11/event8‐02.pdf

[16] West Nusa Tenggara Provincial Health Office . Penguatan program revitalisasi posyandu menuju posyandu keluarga [Strengthening the Posyandu Revitalization Program Towards Family Posyandu]. West Nusa Tenggara Provincial Health Office; 2021. Published June 27, 2021. Accessed February 14, 2023. https://dinkes.ntbprov.go.id/berita/penguatan‐program‐revitalisasi‐posyandu‐menuju‐posyandu‐keluarga/

[17] Mariana MU . Key Success Factors of Community Health Workers Program in Garut, Indonesia (Thesis). Uppsala University; 2019. http://www.diva‐portal.org/smash/get/diva2:1370090/FULLTEXT01.pdf

[18] Indanah I , Jauhar M , Kartikasari F , Karyati S , Rasdiyanah R . Effectiveness of upskilling on improving the attitude of community health volunteers in early detection of childhood stunting. In: Developing a Global Pandemic Exit Strategy and Framework for Global Health Security. Masters Program in Public Health, Universitas Sebelas Maret; 2021:538‐550. 10.26911/ICPHpromotion.FP.08.2021.13

[19] Gadsden T , Sujarwoto S , Purwaningtyas N , et al. Understanding community health worker employment preferences in Malang district, Indonesia, using a discrete choice experiment. BMJ Glob Health. 2022;7(8):e008936. doi:10.1136/bmjgh-2022-008936 PMC9379506 35953209

[20] Perry HB , Chowdhury M , Were M , et al. Community health workers at the dawn of a new era: 11. CHWs leading the way to “Health for All”. Health Res Policy Syst. 2021;19(suppl 3):111. doi:10.1186/s12961-021-00755-5. Published 2021 Oct 12. 34641891 PMC8506098

[21] Fulmer EB , Barbero C , Gilchrist S , et al. Translating workforce development policy interventions for community health workers: application of a policy research continuum. J Public Health Manag Pract. 2020;26(suppl 2):S10‐S18. doi:10.1097/PHH.0000000000001123. Advancing Legal Epidemiology. 32004218 PMC8106979

